# The effects of background music on English reading comprehension for English foreign language learners: evidence from an eye movement study

**DOI:** 10.3389/fpsyg.2023.1140959

**Published:** 2023-06-13

**Authors:** Yankui Su, Meiling He, Rongbao Li

**Affiliations:** ^1^Postdoctoral Station of Psychology, Institute of Psychology, Fujian Normal University, Fuzhou, Fujian, China; ^2^College of Foreign Languages, Fujian Normal University, Fuzhou, Fujian, China

**Keywords:** background music, English reading comprehension, background music preference, eye movement study, music tempo

## Abstract

Based on previous literature, the present study examines the effects of background music on English reading comprehension using eye tracking techniques. All the participants, whose first language was Chinese, were selected from a foreign language college and all of them were sophomores who majored in English. The experiment in this study was a 2 (music tempo: fast and slow) × 2 (text difficulty: difficult and easy) × 2 (background music preference: high and low) mixed design. Both musical tempo and English reading passage were within-subjects factors, and the level of music listening preference was a between-subjects factor. The results showed that the main effect of the music tempo was statistically significant, which indicated that participants read texts more quickly in the fast-tempo music condition than in the slow-tempo music condition. Furthermore, the main effect of the text difficulty was statistically significant. Additionally, the interaction between the text difficulty and music tempo was statistically significant. The music tempo had a greater effect on easy texts than on difficult texts. The results of this study reveal that it is beneficial for people who have a stronger preference for music listening to conduct English reading tasks with fast-tempo music. It is detrimental for people who have little preference for background music listening to complete difficult English reading tasks with slow-tempo music.

## 1. Introduction

It is common for people to listen to music while conducting daily activities in contemporary society. Furthermore, reading a text is always prone to take place with plenty of potential distractions. There are a number of researchers who have investigated the interplay between background music listening and reading comprehension. These studies to some extent provide evidence that reveals the effects of background music on reading comprehension.

Reading, a high-level cognitive performance, plays an important role in our daily life. Also, reading is a significant means of knowledge acquisition. Reading, more or less, happens every day, but not always in quiet surroundings. That is, reading performance may be disturbed by irrelevant sounds. Reading, a complex cognitive task, requires word recognition and semantic integration to acquire information (Cauchard et al., 2012). Music also plays a very important role in today’s society. An experiment performed by Gorn (1982) showed that subjects were willing to choose a specific color of pen if the pen was paired with joyful music instead of displeasing music. Since then, Gorn’s research has become rather popular, and relevant studies of the effects of background music on our daily lives increased. Background music permeates our daily lives. It is probable that people might not perceive the effects of background music when they perform daily tasks, such as driving, eating, reading, working, and even studying. How does background music influence people who listen to it by coincidence or on purpose? There are three kinds of primary findings regarding previous research on the effects of background music. That is, background music may have beneficial, detrimental, or no effect on behaviors.

There are some studies which reveal that background music has a positive effect on task performance. Hall (1952) explored the possible application of music in school education, and he found that students’ comprehension was greatly improved when they listened to music. All the participants were divided into four groups in his study. The four groups of students read a long passage in jazz music, pop music, classical music, and silent conditions. The results showed participants in the classical music condition performed the best among all four conditions. There was no difference between the jazz music condition and silent condition. However, participants listening to pop music performed worst compared with the other three conditions. Additionally, Hurst (2011) investigated the effects of background music on reading comprehension by asking 20 students to finish reading comprehension tests in a classical music condition or non-music condition. He found that participants did a little bit better in the classical music condition than in the silent condition. But the difference between the two conditions was not significantly obvious.

Recently, McDonald (2013) investigated the link between preferred music genre and extroversion on reading performance. The result of his study showed that music could facilitate reading performance if the music which was listened to was what they preferred. On the other hand, conclusions of a negative or null effect of background music on task performance have been drawn by a number of researchers. Etaugh and Michals (1975) carried out an experiment to investigate the effects of background music on reading comprehension. Thirty-two participants were required to finish reading comprehension tests in two conditions: a familiar music condition or non-music condition. The result revealed that there was a main effect for gender differences. Females performed more poorly in the music condition than in quiet surroundings and the performances of males in the two conditions were not largely different.

Perham and Sykora (2012) discovered that performance in silent conditions was better than those both in preferred music conditions and disliked music conditions. In addition, participants performed better with preferred music than with disliked music. However, Roger et al. (2011) examined the effects of background music on reading comprehension using eye tracking techniques. In his study, 24 university students were asked to read four different articles in four different conditions. Participants read while listening to their preferred music, read with music that they did not prefer, read with a recording of noise from a coffee shop, and read in a silent condition. The results revealed that there was no significant difference between the conditions, which was found using the main eye-movement measures for reading. More recently, An-Che and Chen-Shun (2013) found that there was no statistical difference between the test scores and heart rates of participants during reading comprehension and mathematical computation with different kinds of background music.

The technique of eye tracking has unique advantages in exploring the effects of background music on English reading comprehension. For example, the movement of the subjects’ eyes while they read materials can be recorded in real-time using eye tracking, which is more objective and natural than other techniques. Furthermore, high ecological validity of the experiment is ensured if the technique of eye tracking is used. Therefore, the technique of eye tracking is widely applied in the studies of cognition. Moreover, every stage of the effects of background music on task performance can be reflected in the differences among the targets of eye movement that are tracked by an EyeLink. This contributes to exploring the mechanisms of factors driving the effects of background music on reading.

### 1.1. Definition of background music

There have been numerous studies investigating the effects of background music, so the definition of background music should accordingly be set forth. Radocy and Boyle (1988) suggested that background music could be defined as any kind of music which is played while the listener’s primary attention is focused on another task or activity. Similarly, Furnham et al. (1999) held that background music involves music which is played in the workplace, such as factory and offices, accompanying tasks which require varying levels of skill and concentration.

Previous studies have indicated that the effects of different kinds of background music on human activities vary from each other. When background music is a variable in the experiment, factors of the music that should be considered become an important issue for the researchers to think about. Generally speaking, there are three factors that researchers have most investigated regarding background music. They are the type of the music, the music tempo, and the information in the music.

First, the tempo of music is a crucial element in the studies of the effects of background music on cognitive tasks. From the point of view of the musical profession, music with 60 beats per minute is classed as slow-tempo music, whereas music with 120 beats per minutes is regarded as fast-tempo music. Kari (2002) investigated the tempo effects of background music on reading comprehension. He examined the effects of background music on reading business news in a crowded cafeteria environment. Measures were taken in three reading conditions. They were no background music, fast-tempo music, and slow-tempo music. The results indicated that the reading condition notably influenced both the reading measures and the emotional evaluation of the news content. Moreover, the reading rate and efficiency was significantly lower in the slow-tempo music group than in the fast-tempo music group.

Conversely, Thompson et al. (2011) examined the effects of background music on reading comprehension by manipulating variables to create four repeated-measures conditions: slow/soft (110 bpm, 60 dB); slow/loud (110 bpm, 72.4 dB); fast/soft (150 bpm, 60 dB); and fast/loud (150 bpm, 72.4 dB). In their study, tempo and intensity were manipulated using ProTools software (version 7.3). Baseline performance was established by having the subjects complete the reading task in silence. The findings indicated that listening to background instrumental music was most likely to impair reading comprehension when the music was fast and loud. Because of this greater intensity, music listening might consume more of the listeners’ finite attentional resources when it comprised a greater number of auditory events per unit time that were difficult to ignore. By comparison, slow-tempo music might allow for continuous and spontaneous recovery from acoustic interference, permitting simultaneous verbal comprehension even when the music is loud. It was noted that the tempo of music had various effects on English and Chinese reading performance: the faster the background music was, the slower the reading speed. By contrast, Chinese reading speed was faster than that of English reading when it came to faster background music. These experiments reached different conclusions on account of the disparate variables. Additionally, Kari (2002) used no background music, fast music, or slow music as the background music, which could not exclude the effect of the positive mood of the music. In addition, the music material in Thompson et al. (2011) was originally from Mozart’s Sonata for Two Pianos in D major, which might have been familiar to the subjects (Plantinga and Trehub, 2014). To our knowledge, more reliable evidence is necessary to support the ideas obtained in previous studies.

The type of background music is another issue that influences the effects of background music on task performance. Musical genres that have been used in these kind of studies can roughly be classified into five genres: popular music, rock music, jazz music, natural sounds, and classical music. The reason why researchers take these kinds of music as the variables is that they are so common that the majority of people like to listen to them. Numerous researchers have investigated how music genre affected cognitive function. Furnham and Stephenson (2007) demonstrated that performance of reading comprehension, free recall, mental arithmetic, and verbal reasoning tasks while listening to calm music was better than while listening to upbeat music. At the same time, Cassidy and MacDonald (2007) examined the effects of background music and background noise on the task performance of introverts and extroverts. They found that performance of comprehension and free recall were better while listening to slow-tempo music compared with fast-tempo music. Two major categories of study activities were examined in four different conditions of background music genre. The study activities were reading comprehension and mathematical computation. The music conditions were soft music, rock music, heavy metal music, and no music. The results suggested that the performance without background music was relatively poor while the performance during soft music was the best. Music type had a statistically significant effect on test performance. From the perspective of all the previous findings, it is noted that negative or positive effects of background music on reading comprehension probably relies on the characteristics of the music.

The amount of information in the music itself also influences the effects of background music on task performance. The information that exists in the music includes the complexity of the instrumental music and lyrics in the songs. According to neuropsychological research, when an individual listens to music, the brain processes the lyrics and melodies independently (Besson et al., 1998). This supports the notion that not only do these two types of listening compete with each other in having different functions, but they are likely to compete with additional demands on the brain. Similarly, Broadbent (1958) cited the limited capacity model as a framework to explain the negative effects of competitive tasks on concentration. Pool et al. (2003), who were proponents of the limited capacity model, held that attempting to carry out two tasks that draw on inherently limited cognitive resources would work to the detriment of one or both. They demonstrated that trying to complete two tasks simultaneously exceeded a person’s capacity for attention. However, Bourke et al. (1996) argued that the fatal factor was not whether the cognitive capacity was exceeded, but rather that performance declined when both tasks involved processing the same type of information.

### 1.2. Background music preference

Previous studies have focused much attention on the effect of the information in background music on reading comprehension, that is to say, individual differences have often been neglected in previous studies. Huang and Shih (2011) suggested that the influence of background music on daily activities was mostly determined by personal preference. According to educational psychology, the habit of studying while listening to music is regarded as a type of learning style. A great deal of studies of music preferences were carried out in the last century (Heyduk, 1975; Steck and Machotka, 1975), and there have been several good reviews and theoretical integrations which, though vastly different in breadth, were all helpful for a better understanding of preference (Meyer, 1956). Deems (2005) studied the effects of background music on reading comprehension by examining two groups of participants, those who preferred studying while listening to music and those who did not. The conclusions showed that the former group performed better than the latter. Because of the inconsistent findings, the effects of background music preference requires further investigation.

In short, the relationship between reading and listening to music has been discussed in many studies and from a variety of perspectives. However, this research has brought about diverse and conflicting results which indicate that the effects of background music is very complicated and tough to investigate. The purpose of the present study is to reveal the effects of music listening more accurately. The present study will re-examine the interplay between music and text difficulty by taking individual factors concerning music preference into consideration.

## 2. Methods

### 2.1. Participants

A total of 130 participants with normal visual and hearing capabilities were recruited from the College of Foreign Languages, Fujian Normal University. All were willing to participate in the experiment. They were given a gift for their attendance. They were sophomores majoring in English and they were paid for their participation after the experiment. There were 100 participants who finished the music preference questionnaire. The principal experiment was finished by 77 participants who were selected from the 100 participants. It must be noted that the other 30 subjects were assigned to take part in a pilot study conducted to verify the difficulty and familiarity of the reading test passages for the principal test. The 30 subjects in practice were not involved in the principal experiment. Seventy-seven students participated in the principal experiment. None of them were exposed to any portion of the test materials which were employed in this study. However, during the last part of the experiment, only the data of 60 participants were analyzed because of missing or excluded data.

### 2.2. Instruments

The instruments used in this study included a background music preference questionnaire, which was utilized to group the participants into two parts. One group had a high level of background music listening preference and the other had a low level of background music listening preference. An Eyelink 1,000 eye tracker (SR Research Ltd) used to record the trace of the reading was also adopted in this study. The sampling rate was 1,000 Hz and the eye movement trajectory was recorded every millisecond. The English reading materials appeared on a 17-inch Dell LCD monitor which was 75 centimeters away from the participants and had a resolution of 1,024 × 764. Although the participants read the passages with their natural eyes in a natural way, only the movement of the left eye was monitored by the eye tracking technique. In addition, a post-test questionnaire was used to make sure that all the participants were unfamiliar with the English test materials and music stimuli. Additionally, SPSS 25.0 was used to analyze the data. Furthermore, two computers were applied in this study, one of which was a desktop used as the display screen and the other one was to play the music stimuli.

### 2.3. Materials

The materials were composed of English reading test materials, music stimuli, a music preference questionnaire, and a post-test questionnaire.

#### 2.3.1. English Reading test materials

The reading test materials for the principal experiment differed in test difficulty. The more difficult ones were selected from reading passages in Science magazine, and the simpler ones were from reading comprehension tests in practice tests of the College English Test Band Four in China. There were five passages each with about 70 English words from each difficulty level. The number of the passages was ten, two of which were for the practice experiment, and eight were for the principal experiment. In order to distinguish the difficulty of those selected passages, a pilot study was conducted using the 30 participants who did not take part in the principal experiment. All the reading test materials were expositions with one judgment question. The function of the question here was to remind the participants to read the passages carefully. The question was a main idea question. All the English reading materials were assessed by a college teacher who taught participants English writing. Participants’ familiarity with the reading passages was also examined. The evaluation of readers’ familiarity with the reading materials was made on a 5-point scale, where 1 meant that readers were not familiar with the reading materials at all and 5 meant that the readers were very familiar with the reading materials. The difficulty of the reading materials was also evaluated on a 5-point scale, where 1 referred to the easiest reading passage and 5 represented the most difficult reading passage. Estimations of the difficulty of the reading materials and readers’ familiarity with the experimental reading materials are shown in Table 1.

**Table 1:** Descriptive statistics of the familiarity and difficulty of the reading materials.

Materials	Familiarity		Difficulty	
	*M*	SD	*M*	SD
Reading 1	1.03	0.18	1.07	0.25
Reading 2	1.17	0.46	1.03	0.18
Reading 3	1.1	0.30	1.07	0.25
Reading 4	1.13	0.35	1.10	0.31
Reading 5	1.07	0.25	4.60	0.68
Reading 6	1.20	0.41	4.83	0.38
Reading 7	1.03	0.18	4.93	0.25
Reading 8	1.07	0.25	4.90	0.31

#### 2.3.2. Background music stimuli

There were three kinds of music which were applied in this study. The first one was selected from the prelude of Bach’s Piano Sonata in G. The other two were also based on the same piece of music. These three kinds of background music were exactly the same except for the music tempo. The tempo of the original music is 72 beats per minute. The original music was applied in the practice experiment. The other two versions were used in the final experiment. The tempo of the last two were 60 and 120 beats per minute, respectively, of which the faster version was called fast background music and the slower version was called slow background music. Familiarity with the three kinds of background music was examined on a 5-point scale, where 1 meant participants did not know the music at all and 5 meant that participants were very familiar with the music. The music familiarity evaluation is shown in Table 2.

**Table 2:** Descriptive statistics of the music familiarity.

Music tempo	*M*	SD
Original tempo	1.18	0.469
Fast tempo	1.03	0.181
Slow tempo	1.08	0.334

#### 2.3.3. Background music preference questionnaire

The background music preference questionnaire was used to evaluate the level of music preference of the participants. This questionnaire included 13 items, each with five levels. In order to make sure the background music preference questionnaire was effective, 100 participants finished the questionnaire. Twenty-three of the 100 participants did not attend the principal experiment. The final credibility of the questionnaire was 0.901, which was achieved using the SPPS 25.0 for Windows.

#### 2.3.4. Post-test questionnaire

It was required that none of the test materials in this study had been previously read or completed by participants, so a post-test questionnaire was needed. Participants were asked to complete a short exit questionnaire, inquiring whether they were familiar with the English reading materials and the background music stimuli. It also required participants to evaluate the difficulty level of the English reading materials.

### 2.4. Experimental design

The experiment was a 2 (music tempo: fast and slow) × 2 (English reading text difficulties: difficult and easy) × 2 (music preferences in reading: high and low) mixed design. Both music tempo and English text difficulty were within-subjects factors, and the musical preference during reading was a between-subjects factor.

### 2.5. Research procedure

There were three steps in the research procedure, which included the pilot study, the selection of the participants, and the principal experiment.

#### 2.5.1. The pilot study

The study was held in a sound-attenuated classroom with 30 participants. All the participants were asked in Chinese to verify the difficulty of the English reading materials and their familiarity with the English reading materials. The test paper was composed of 14 English reading passages, each of which had a judgment question. The English reading materials contained seven difficult passages and seven easy passages. The degree of text difficulty was evaluated by an English teacher from the Foreign Language College first. After reading each passage, the participants had to evaluate the difficulty and their familiarity with each English reading article from one to five points. The higher the score they gave, the more difficult the reading passage was and the more familiar they were with the English reading passage. The participants were required to finish the test paper within 30 min.

Thereafter, all the test papers were collected to analyze the results. The degree of difficulty of the 14 English reading passages and the participants’ familiarity with the reading passages were counted and compared. Also, the answers to the true-false questions were examined. As a result, it turned out that all the participants were unfamiliar with the English reading materials. In addition, the reading materials were grouped into two parts according to the scores that all the participants gave. Furthermore, 80% of the answers to the judgment questions were correct, which to a great extent suggested that the participants read the materials carefully. Finally, 10 of the 14 English reading materials were chosen for the final experiment. Five English reading passages were difficult, and the other five were relatively easy. The difficult ones were of the same difficulty level. Similarly, the simple ones also shared the same level of difficulty. Finally, all the English reading materials selected were suitable for the principal experiment.

#### 2.5.2. The selection of participants

One hundred participants were asked to complete the background music listening preference questionnaire within 10 min. According to the result of the questionnaire, 77 of the 100 participants were selected to attend the final experiment. The 77 participants were classified into two groups on the basis of the scores that they received in the background music listening preference questionnaire. Generally, participants with higher scores had stronger preferences for music listening and were grouped into the high level of preference for music listening during reading category. At the same time, participants who showed less interest in listening to music during reading were grouped into the low level category.

#### 2.5.3. The principal experiment

All 77 participants received a list of English words and expressions which would appear in the final experiment. The participants were requested to remember the word list so as to avoid failure in reading because of poor understanding of words and expressions. The experimenter would check that each participant had a good understanding about the word list so that the participant could attend the final experiment. In the principal experiment, each participant was tested individually. They were instructed to sit in front of the desktop and put on earphones. The experimenter played music to the participant and asked whether they were satisfied with volume of the music. The volume of the music was regulated according to the participants’ habits. At the very beginning, the participants were told to rest their chin on a holder and read the instructions on the screen carefully and silently. The participants were encouraged to voice any doubts if they had any question about the procedure of the final experiment, and the experimenter would clarify the key points. The participants were asked to press any button on the keyboard to enter the calibrate stage as soon as they were clear about the experimental procedure.

The calibration was validated after the participant looked accurately at five different points which appeared one after another in five varying positions on the screen. Prior to the presentation of each stimulus, a black dot would appear on the middle left of the screen to guide the participants’ eye movements. After valid calibration, two practice reading passages were presented to familiarize the subjects with the procedure. After that, there was the critical session. The critical session was divided into two periods: one was the fast music period, in which four English reading passages were presented. The difficulty sequence of the four reading passages was: difficult, easy, difficult, and easy. The other period was the slow music period. The pattern of the four reading passages in the slow music condition was the same as that of the fast music period. The participants were directed to read the passages at their own personal pace. When the critical session was completed, participants were given a post-test questionnaire to ask them about their familiarity with the reading materials and the music stimuli. Participants were also queried about the perceived text difficulty. The whole procedure lasted about 25 min.

#### 2.5.4. Data analysis procedure

Subjects were all engaged during the experiment and grasped the meaning of the reading passages, with a mean correct rate of more than 84% for the judgment questions. According to the established practices, some data were trimmed before the final analysis (Rayner, 1988). Specifically, (1) the eye movement data from subjects were discarded on account of some serious data missing; (2) fixations that were shorter than 80 ms or longer than 1,200 ms were eliminated in that excessively short fixations could not be a sign of mental processing and unduly long fixations were usually due to equipment errors; (3) a single test where the count of fixations was less than 4 was discarded; (4) outlying data were eliminated; and (5) data were excluded where the tracker failed to log the trajectory of the eye. The data which lay three standard deviations from the mean were regarded as outliers. The removal of this data was 22% of the total. The eye movement data of 60 participants were exported from Data Viewer (SR Research Ltd) and processed in Microsoft Excel and IBM SPSS Statistics 25. Primarily, three-factor analysis of variance was carried out to reveal the effects background music exerted on reading performance; tests of within-subjects effects were added to show the significance of the facilitation and interference of music tempo and the text difficulty. Taking background music listening preferences into consideration, a mixed repeated-measures ANOVA was conducted to examine the main effects and interactive relationships among the levels of the preference for listening to music while reading, text difficulty, and music tempo.

Five eye-movement measures were collected in this experiment. They were average saccadic amplitude, fixation count, total fixation duration, saccade count, and pupil size mean. The whole reading passage in a single test was treated as the area of interest. We were interested in the reading duration of the whole passage. The definitions and ways of measurement were as follows: (1) Average saccadic amplitude. This is the average size (in degrees of visual angle) of all selected saccades. The amplitude of saccade is influenced not only by the difficulty of the reading materials but also by the length of words in the texts (Phillips and Edelman, 2008). A larger saccadic amplitude means participants have grasped more information in the fixation before the saccade happens. (2) Fixation count. This is also called the total number of fixations. It computes the number of times that the eyes view the interest area. It indicates the cognitive burden of the material in the interest area. More fixations in the interest area means a heavier load of cognitive processing. (3) Total fixation duration. This is equivalent to what is named total viewing time by some academics. As the name indicates, total fixation duration is calculated as the summation of the length of all the fixations in the interest area. (4) Saccadic count. This refers to the total number of saccades in the interest area. (5) Pupil size mean. Changes in pupil size can be regarded as a good indicator of cognitive load changes in speech processing.

The reason why we choose the five eye-movement indexes as the measurement in the present study was decided by the benefit of those eye-movement measurements. First, the higher the fixation count is, the lower the efficiency of task performance. That is, the fixation count number reveals the efficiency of the task performance. However, the increasing amount of the fixation count in a specific interest area can also be explained in that more attention is being paid to it. The second measurement is total fixation duration. There are two explanations for a longer total fixation duration. On one hand, when it is more difficult to extract information from the reading materials, a longer total fixation on the reading materials will happen; on the other hand, people will fixate something longer if it is interesting. Third, the saccade count number of specific areas represents the research process of this area. The higher the saccade count is, the longer it takes for information processing. Next, a larger average saccadic amplitude can be explained by the interest that people have in the next interest area. Lastly, it is concluded that cognitive load leads to larger pupil size. Reading is a complex cognitive task, which places demands on cognitive resources. Difficult English reading comprehension requires more cognitive resources than easy ones.

## 3. Results

The data have been obtained from the experiment. The data of total fixation duration and pupil size means were chosen for analysis. Data analysis was divided into two parts. One is the descriptive analysis, and the other is the main effects and interactive effects of the three variables in this study.

### 3.1. Descriptive statistics

#### 3.1.1. Total fixation duration

As is shown in Table 3, participants spent more time on the difficult reading passages. However, the time spent in the fast background music condition was less than in the slow background music condition. In addition, the participants with high background music listening preferences spent less time on the reading comprehension compared with participants with low listening preferences.

**Table 3:** Descriptive statistics of total fixation duration.

	*p*	*A*	*M*	SD	*N*
B1	G	A1	44863.25	14399.90	30
		A2	25637.77	6925.03	30
	D	A1	47140.88	15551.83	30
		A2	27507.23	9043.33	30
B2	G	A1	46123.58	13958.97	30
		A2	32897.48	11790.56	30
	D	A1	52493.97	16533.15	30
		A2	34963.73	12339.27	30

*p* means background music listening preference; G refers to a high level of background music.

Listening preference while reading; D refers to a low level of preference for background music.

Listening while reading; A is the text difficulty; A1 is the difficult reading materials; A2 is the easy.

Reading materials; B1 refers to fast background music; B2 is the slow background music.

#### 3.1.2. Pupil size mean

As is shown in Table 4, the mean pupil size of participants with high music listening preferences in the fast background music condition were obviously smaller than that of participants with low levels of music listening preference. However, in the slow-tempo music condition, the pupil size of participants who reported that they had a stronger preference for background music listening while reading was larger than those who had lower levels of preference for reading with music.

**Table 4:** Descriptive statistics of pupil size mean.

	*p*	*A*	*M*	SD	*N*
B1	G	A1	959.09	325.55	30
		A2	956.05	324.85	30
	D	A1	1034.17	295.19	30
		A2	1027.95	295.95	30
B2	G	A1	926.94	308.50	30
		A2	941.49	315.98	30
	D	A1	646.37	194.37	30
		A2	670.53	183.48	30

### 3.2. Main effect analysis and interactive effect analysis

#### 3.2.1. Total fixation duration

For the total fixation duration, the within-subjects effects are described in Table 5 and show that the main effect of text difficulty was significant, *F* (1,58) =238.79, *p* = 0.00 < 0.05, and the main effect of music tempo was observed, *F* (1,58) =44.99, *p* = 0.00 < 0.05. What is more, there was an interactive effect between text difficulty and music tempo. Table 6 report the tests of between-subjects effects and show that there was no main effect in background music listening preference while reading, *F* (1,58) =1.12, *p* = 0.29 > 0.05. There was no interactive effect between the three factors including text difficulty, music tempo, and the levels of preferring to listening to music while reading.

**Table 5:** Tests for within-subjects effects of total fixation duration.

	SS	df	MS	*F*	*p*
A	18173674497.06	1	18173674497.06	238.79	0.00
A * P	83271642.33	1	83271642.33	1.09	0.30
B	1706074718.00	1	1706074718.00	44.99	0.00
B * P	69000360.81	1	69000360.81	1.82	0.18
A * B	246207629.40	1	246207629.40	8.84	0.00
A * B * P	56919586.00	1	56919586.00	2.04	0.15

**Table 6:** Tests for between-subjects effects of total fixation duration.

	SS	df	MS	*F*	*p*
Intercept	364169805219.03	1	364169805219.03	690.54	0.00
P	593813792.26	1	593813792.26	1.12	0.29
Error	30587047494.57	58	527362887.83		

#### 3.2.2. Pupil size mean

For the pupil size mean, the results of all the main effects of all the three factors are shown in Table 7. Specifically, the main effect of the text difficulty was not significant, *F* (1,58) =0.85, *p* = 0.35 > 0.05. Additionally, there were no interactive effects between the text difficulty and the level of preference for listening to music while reading. However, the main effect of the tempo of background music was significant, *F* (1,58) =101.25, *p* = 0.00 < 0.05. The interactive effect between the tempo of the background music and the levels of preference for listening to music while reading was also significant, *F* (1,58) =78.77, *p* = 0.00 < 0.05. But the interactive effect between the two within-subjects factors was not significant, *F* (1,58) =2.02, *p* = 0.16 > 0.05. There were no interactive effects between the three factors, *F* (1,58) =0.14, *p* = 0.70 > 0.05. Table 8 report the tests of between-effects of the pupil size mean. There was no main effect on the pupil size mean, *F* (1,58) =2.08, *p* = 0.15 > 0.05.

**Table 7:** Tests for within-subjects effects of pupil size mean.

	SS	df	MS	*F*	*p*
A	3255.51	1	3255.51	0.85	0.35
A * P	155.27	1	155.27	0.04	0.84
B	2351815.30	1	2351815.30	101.25	0.00
B * P	1829733.84	1	1829733.84	78.77	0.00
A * B	8632.38	1	8632.382	2.02	0.16
A * B * P	613.32	1	613.328	0.14	0.70

**Table 8:** Tests for between-subjects effects on pupil size mean.

	SS	df	MS	*F*	*p*
Intercept	192384758.97	1	192384758.97	652.04	0.00
P	613684.65	1	613684.65	2.08	0.15
Error	17112749.64	58	295047.40		

## 4. Discussion

In response to the belief that reading comprehension is impaired by the tempo of background music, and to the suggestion that reading performance is only enhanced when the participants tended to listen to preferred music while reading, the present study was conducted to assess the effect of the tempo of music and the level of preference for music listening while reading on English reading comprehension.

### 4.1. The effect of music tempo

The main effect of the music tempo in this study was statistically significant. In particular, the performance in the fast-tempo music condition was better than that in the slow-tempo music condition, which was inconsistent with previous studies (Kari, 2002; Thompson et al., 2011). Music of 120 beats per minute was chosen as the fast music in the present study. According to the arousal and mood hypothesis, music listening affects arousal and mood, which in turn impacts task performance of cognitive skills. The explanation of this result can be concluded as follows: task performance could be influenced by mood and emotions, and the cognitive-motor benefits are associated with enhanced mood and heightened arousal. Furthermore, mood and arousal can be enhanced by music of 120 beats per minute, so the music of 120 beats per minutes used in this study, being fast-tempo music, could facilitate English reading comprehension.

### 4.2. The effect of background music listening preference

In the eye movement measurements, participants with a higher level of background music listening preference did better in task performance than those of a lower level of preference, which contradicted with previous studies (Stacey and Gerald, 2010). Previous studies have shown that the harmful effect of background music on reading comprehension was more pronounced for students who had a stronger preference for music listening while learning and most students who showed a preference for music listening while studying performed more poorly with background music than those who preferred to study without background music. There are several possible explanations for the result of this study. According to the affective filter hypothesis, motivation, anxiety, and self-confidence are three factors that impact second language learning. self-confidence can enable better language learning. The preference for music listening while studying is good for foreign language learning. Good task performance in English reading comprehension can be achieved by having a high preference for music listening, because the efficiency of second language learning can be influenced by the affective factors. Participants who had a stronger preference for music listening while studying were accustomed to reading with music, and listening to music while reading could ease stress and anxiety to make them happier and more willing to read English texts, which could also facilitate English reading comprehension. But the reverse effect might occur for those students who prefer reading in quiet surroundings.

### 4.3. The interaction between music tempo and text difficulty

Text difficulty had a main effect in the eye-movement measurements, which meant that the more difficult the reading material was, the more time or strength would be put on it to process it. What is more, the interactive effect between text difficulty and music tempo was statistically significant. That is, the effect of music tempo was affected by the text difficulty. The music tempo had a greater impact on easy texts than on difficult texts. This effect was also revealed in the eye measurements. The explanation for this effect was that the difficult texts in this study were difficult for the participants, so the participants did not read them carefully, but when the easy texts were exposed to the participants, they were more willing to read the simple texts carefully, so the tempo of the music had a greater influence on the reading comprehension of easy texts compared with complex texts.

### 4.4. The interaction between music tempo and background music preference

The interactive effect between background music listening preference and music tempo was statistically significant, which was shown in the eye measurements of pupil size mean. The effect of music tempo was affected by the preference for music listening. Background music tempo had a greater influence on the reading of participants with a lower level of music listening preference than that of participants with a higher level of preference. Studying with background music is a kind of habit. This kind of habit might to some extent reduce the effect of music tempo, so it can be concluded that, in general, the effects of music tempo would be diminished because of the habituation effect. Moreover, the pupil size of participants who had a stronger preference for reading with music in fast-tempo music surroundings was smaller than those of participants of a lower level of preference. However, in the slow-tempo music condition, pupil size was larger for the high level of preference participants than the lower ones. What was the underlying reason for this kind of interaction? The index of pupil size can reveal one’s tension of pressure. The smaller the pupil size, the more pressure the participants may bear. In the fast-tempo music condition, a higher level of preference for reading with music listening can make participants more relaxed than a lower level of preference, and their pupil size mean is also smaller. However, in the slow tempo music condition, the effect of music tempo played a more important role than the effect of music listening preference.

## 5. Limitations

Similar to other studies exploring background music and cognitive tasks, there are also some limitations to this study. First, the effect of background music on English reading comprehension is also affected by the ability of the participants in English reading comprehension. However, the recruitment of participants for the experiment was more difficult than previously planned, which resulted in an uneven and small sample size. Actually, the recruitment of the participants for the experiment was based on their score from the College English Test Band Four, so the ability of English reading comprehension of all the participants was not scientifically similar. What is more, because of the fact that there were more females than males in the foreign language college, the number of male participants was smaller than that of female participants. Previous studies have found that the effect of background music on reading may be on account of gender differences (Kari, 2002; Stacey and Gerald, 2010).

## 6. Conclusion

To sum up, this study investigates the effects of background music on English reading comprehension from three perspectives: text difficulty, music tempo, and background music listening preference. This study was implemented on the basis of previous studies, but it differs from previous research. Firstly, the present study is the first attempt at examining the influence of background music on English reading comprehension from three perspectives, which has scarcely been covered in previous studies. Secondly, from the perspective of individual differences, the factor of background music preference was used in this study. Lastly, the effect of background music on task performance is also regulated by the difficulty of the task. As the results of the study are discussed above, the conclusion can be drawn below.

People who had a preference for studying while listening to music performed better in the task performance than those who showed little or did not show preference for music listening while reading. This is one of the main findings of the study; thus, students who do not have a strong preference for reading to music should not often perform reading tasks with background music.

Furthermore, the music tempo also influenced the reading activities. Fast-tempo music could improve English reading comprehension. The population of EFL learners in China becomes larger and larger, so the topic of how to improve English language teaching and learning becomes particularly crucial for both teachers and students. Music is an important tool for foreign language teaching and learning. Fast-tempo music can activate students’ arousal and mood so that students may have a good mood and be more willing to engage in the activities of English classes. Therefore, teachers can apply fast-tempo music to English language classes.

In addition, the interplay between the factor of background music preference and music tempo is clear in this study. People with a high level of background music listening preference perform better than those of a low level of background music listening preference. On account of this result, it is wise for people who report that they have a stronger preference for background music listening to perform reading tasks in a music condition.

Lastly, the findings on music tempo indicate very clearly that it is necessary to study the effects of the structural properties of the background music in detail. The inconsistent results of previous research may be due to the factors that are taken into consideration. In the investigation of the effects of background music on reading comprehension, there are three main factors that should be examined. They are the background music, individual differences, and the difficulty or properties of the reading tasks. The results of this study could be further investigated by consideration of all these factors. It is beneficial for people who have stronger preference for music listening to conduct English reading tasks with fast-tempo music. It is detrimental for people who have little preference for background music listening to complete difficult English reading tasks with slow-tempo music.

## Data availability statement

The raw data supporting the conclusions of this article will be made available by the authors, without undue reservation.

## Ethics statement

The studies involving human participants were reviewed and approved by the Ethics Committee of Fujian Normal University. The patients/participants provided their written informed consent to participate in this study.

## Author contributions

YS and MH made substantial contributions to the conception, design, acquisition of the data, and analysis and interpretation of the data. RL developed the theory, verified the analytical methods, and supervised the findings of this work. All authors contributed to the article and approved the submitted version.

## Funding

This work was supported by China Postdoctoral Science Foundation (2019M652239).

## Conflict of interest

The authors declare that the research was conducted in the absence of any commercial or financial relationships that could be construed as a potential conflict of interest.

## Publisher’s note

All claims expressed in this article are solely those of the authors and do not necessarily represent those of their affiliated organizations, or those of the publisher, the editors and the reviewers. Any product that may be evaluated in this article, or claim that may be made by its manufacturer, is not guaranteed or endorsed by the publisher.
